# *Populus* Callus Cell Lines: A Novel Source of Extracellular Vesicles with Nanocarrier Potential

**DOI:** 10.3390/cimb47121015

**Published:** 2025-12-05

**Authors:** Miguel Rito, Sandra Caeiro, Pedro Rosa, Cristina Azevedo, Sandra Correia

**Affiliations:** 1 Centre for Functional Ecology, TERRA Associate Laboratory, Department of Life Sciences, University of Coimbra, Calçada Martim de Freitas, 3000-456 Coimbra, Portugal; miguel.rito@student.uc.pt; 2 InnovPlantProtect CoLAb, Estrada de Gil Vaz, 7350-478 Elvas, Portugal; s.caeiro@iplantprotect.pt (S.C.); pcrosa@iplantprotect.pt (P.R.); cazevedo@iplantprotect.pt (C.A.)

**Keywords:** callus, extracellular vesicles, RNA-loading, RNA-delivery, nanocarrier

## Abstract

Developing sustainable and eco-friendly approaches to plant propagation, development, and protection is a common goal for the scientific community. Plant cell culturing enables us to obtain plant clones and produce biomolecules under controlled conditions. The same principle can be applied to the harvesting of extracellular vesicles (EVs). These nanosized structures are key players in cell communication and stress response by carrying, protecting, and delivering important biomolecules. Raising interest in the scientific community, EVs have been successfully tested as nanocarriers for therapeutics and biotechnology. However, despite their potential, there remains a gap in research on scalable, reliable sources for EV production. Our goals were to optimize EV production and isolation from induced poplar callus cell lines (*Populus tremula* × *P. alba*) and load these with RNA to validate their functionality as nanocarriers. We were able to isolate 2.5 × 10^10^ EVs/g, highlighting the potential for these lines to be mass-produced. Furthermore, RNA loaded into EVs through electroporation was internalized into *Botrytis cinerea* hyphae, reassuring their potential in protecting and delivering cargo. Our findings contribute to EV characterization and demonstrate that RNA delivery through EV transport could be a safe and effective method for future EV-based technologies in plant protection.

## 1. Introduction

Callus induction in vitro, or callogenesis, represents a well-established technique in plant biotechnology for generating undifferentiated plant tissue under controlled conditions [1]. This method not only enables the continuous propagation of plant material independent of seasonal or environmental constraints, when the induced callus has morphogenic potential, as in indirect organogenesis [2] or somatic embryogenesis processes [3], but also serves as a powerful platform for the large-scale production of valuable metabolites and biologically active compounds [4].

In recent years, plant callus cultures have also emerged as a promising source of extracellular vesicles (EVs) [5,6,7,8,9], structures with an elevated biotechnological potential due to their capacity for drug delivery [10]. Most current studies rely on mammalian or limited plant systems, which often face challenges in yield, consistency, and cost-effectiveness [11]. Identifying alternative biological platforms capable of producing EVs at a larger scale is therefore critical to unlocking their full translational potential [12,13].

EVs are nano-sized structures with an average diameter of 80–150 nm and a bilipid membrane that enables them to carry and protect their contents [14]. They are known to transport a wide range of biomolecules, including RNA, proteins, and secondary metabolites [15]. These stand out due to their intrinsic value in molecular studies and applications [16]. Currently, EVs are a hot topic in the development of novel methods in medicine [17], mainly due to their ability to naturally transport biomolecules [18] (e.g., RNA) and their lower toxicity, as they are naturally derived [19]. Some works have already focused on the impact that callus-produced EVs could have in therapeutics [7].

Given the scalability, reproducibility, and manipulability of in vitro systems, callus-derived EVs offer exciting potential for applications in plant science, biotechnology, and nanomedicine [5,9]. However, the use of callus cultures, particularly from woody species such as *Populus*, for EV production remains underexplored and warrants further investigation.

Currently, directives from European authorities aim to reduce the use of chemical pesticides in crop protection and increase the use of more sustainable, environmentally friendly alternatives [20]. A current technique being explored in crop protection is the use of interfering RNA (RNAi), specifically small interfering RNAs (siRNAs) [21]. siRNAs can regulate gene expression by silencing specific target genes in plants or in invading pathogens [22]. Ledprona is an example of an RNAi-based commercial product in the US, where a double-stranded RNA is used against the potato beetle [23]. Although RNAi is an emerging and potentially highly effective technique for plant protection, it still has some issues, such as delivery and instability, particularly the rapid degradation of RNA under environmental conditions [24]. Therefore, their effectiveness could be greatly improved by using nanocarriers such as EVs, which could enhance targeted delivery, RNA stability, and uptake efficiency [25]. Although not EV-based, Bioclay, a formulation of a clay nanostructure that delivers and protects RNA, is an example of what EVs could improve in RNAi-based techniques [26].

*Botrytis cinerea*, commonly known as grey mould, infects a wide range of plant hosts, from fruits to vegetables [27]. Previous studies showed that *B. cinerea* spores readily take up RNA during germination [28], making them an ideal choice for RNA uptake assays.

In this work, we characterize EVs in poplar callus and show that this system is a viable, clean, and valuable source of extracellular vesicles, with the capacity to deliver RNA into fungal cells, filling the gap as a scalable and reliable system for EV production for future EV-based technologies.

## 2. Materials and Methods

### 2.1. Callus Induction and Maintenance

For callus induction, poplar (*Populus tremula* × *P. alba*) leaves from in vitro-established plantlets were used. Leaf segments from in vitro plants were cut within the midrib (1 cm × 1 cm) and punctured in five locations. These were placed in Petri dishes with MS medium [29] supplemented with 30 g/L of sucrose, 7 g/L agar, 1 mg/L 2,4-D (2,4-dichlorophenoxyacetic acid), and 0.05 mg/L BAP (6-benzylaminopurine), and the pH was adjusted to 5.7. The media was autoclaved at 121 °C for 20 min. The leaf segments were incubated at 24 °C in the dark for 17 weeks, with subcultures every 4–6 weeks until a proliferating callus was formed. Afterwards, the induced callus was harvested and placed in fresh MS medium as described above.

### 2.2. Extracellular Vesicles Extraction

Ten grams of callus were harvested and placed on a needleless syringe, filled with VIB (Vesicle Isolation Buffer −20 mM MES, 2 mM CaCl_2_, and 0.1 M NaCl, pH 6.0) [30]. The buffer was filtered through a 0.45 μm Nylon filter (Sterile PES Syringe Filter, Fisherbrand™, Hampton, NH, USA). The buffer was carefully infiltrated by slowly pulling and releasing the plunger. Then the callus was transferred into a syringe fitted with a needle and placed in a 50 mL centrifuge tube. The samples were then run at 900× *g* for 10 min (4 °C), and the apoplastic washing fluid (AWF) was collected. To pellet the EVs, the differential centrifugation method was used [31]. The AWF was centrifuged at 2000× *g* for 20 min, followed by 10,000× *g* for 30 min, at 4 °C. The pellet was discarded, and the supernatant filtered through a 0.45 μm Nylon filter. Then, the supernatant was moved into ultracentrifuge tubes (13.2 mL open-top thinwall, Beckman Coulter, Brea, CA, USA) and centrifuged at 100,000× *g* for 80 min (swinging-bucket rotor SW 41 Ti, L-100 XP centrifuge, Beckman Coulter, Brea, CA, USA). The resulting pellet was washed with fresh buffer and ultracentrifuged a second time at 100,000× *g* for 80 min, at 4 °C. The pellet was resuspended as follows: in 20 μL of VIB for Transmission Electron Microscopy (TEM), 1 mL of VIB for Nanoparticle Tracker Analysis (NTA), and 100 μL of VIB for the loading assays.

### 2.3. Transmission Electron Microscopy

For Transmission electron microscopy (TEM), paraformaldehyde was added to the EV-resuspended samples to a final concentration of 4% (*v*/*v*) and stored in the fridge for up to a week. Samples were transferred to Formvar- carbon coated grids for 5 min and contrasted with uranyl acetate (2%; *w*/*v*) for 1 min. Observations were carried out using a Tecnai G2 Spirit BioTwin electron microscope (FEI, Hillsboro, OR, USA) at 100 kV.

### 2.4. Nanoparticle Track Analysis

The Nanoparticle track analysis (NTA) samples were kept at −80 °C until analysis. Samples were analyzed using Nanosight (Malvern Pananalytical, Malvern, England). EV concentration was adjusted to the initial volume and weight for each sample. The EV size average, mode, standard deviation, and D10/50/90 (percent of the particles are equal to or smaller than) were included in the results.

### 2.5. In Vitro Transcription of Fluorescent RNA

A double-stranded RNA complementary to exons 13 and 14 of wheat Phytoene Desaturase PDS1 (dsPDS) was selected and generated through in vitro transcription (IVT). cDNA from wheat cv. Morocco was synthesized using the SuperScript™ IV One-Step RT-PCR System (Thermo Scientific, Waltham, MA, USA), and two pairs of gene-specific primers (Table 1) were used to amplify a 219 bp segment containing a T7 RNA polymerase promoter at either the 5′ or the 3′ end, respectively. Each segment was cloned into *E. coli* DH5α and sequenced (outsourced to STAB VIDA, Caparica, Portugal). Plasmids containing each segment were purified using NZYMiniprep (NZYTech, Lisbon, Portugal), DNA templates for IVT were amplified by PCR with Phusion High-Fidelity DNA polymerase (Thermo Scientific, MA, USA), and purified using NZYGelPure (NZYTech, Lisbon, Portugal). To produce fluorescently labelled dsPDS (dsPDS-F), Fluorescein RNA Labelling Mix (Roche, Basel, Switzerland) was used according to the manufacturer’s protocol and Hamby et al., 2020, and stored at −80 °C until further use [32]. Integrity of dsPDS-F was visualized by running on a 1% TAE gel with commercial bleach at 1.5% *v*/*v* [33].

### 2.6. Loading Assays

The samples for loading assays were kept at −80 °C. Several methods from previous experiments were used to load the obtained EVs with the fluorescent RNA (100 ng/µL), but electroporation, sonication, and co-incubation were chosen. Before electroporation, a buffer was freshly prepared containing 1.15 mM potassium phosphate (pH 7.2), 25 mM potassium chloride and 21% (*v*/*v*) OptiPrepTM (Iodixanol) (STEMCELL Technologies Inc., Vancouver, BC, Canada). EV solution and electroporation buffer were mixed in a 1:1 ratio and transferred to 2 mm electroporation cuvettes. The samples were electroporated at 400, 200, and 100 Volts (Bio-Rad GenePulser XCell Electroporation System, Bio-Rad Laboratories Inc., Hercules, CA, USA) for 5 ms and incubated in the dark at 37 °C for 30 min [34]. Sonication was performed according to the protocol described by Kim and colleagues (2016) [31]. Six cycles of 30 s on/off for 3 min were applied, with 2 min on ice incubation after each cycle, followed by incubation in the dark at 37 °C for 30 min. For co-incubation, samples were left for 4 h at room temperature with mild shaking. For the freeze–thaw method, 3 cycles of −80 °C to RT, each for 30 min, were performed, followed by incubation in the dark at 37 °C for 30 min. To remove the unloaded RNA, 50 U of Micrococcal Nuclease (MNase, New England Biolabs, Ipswich, MA, USA) was used. To confirm that the EVs were loaded with RNA, samples were observed under a Zeiss LSM 710 Confocal Microscope (Carl Zeiss AG, Oberkochen, Germany). To calculate the loading efficiency between treatments, the green fluorescence-positive area was measured through ImageJ/Fiji Version 1.54p [35]. The fluorescent pixels were counted and plotted as a percentage of the total imaged area. The results were analyzed in GraphPad Prism (version 9, Dotmatics, Boston, MA, USA) using one-way ANOVA followed by Tukey’s post hoc test for statistical comparison between each loading assay. *p*-values less than or equal to 0.05 were considered significant, and the differences were represented by different superscript letters.

### 2.7. Uptake Assays

RNA-loaded vesicles were used for this assay. *Botrytis cinerea* was grown in V8 media [36] with 7 g/L agar, and the pH was adjusted to 6.0. The media was autoclaved at 121 °C for 20 min. Then, the cultures were kept at 24 °C in a 16/8 h light cycle until sporulation (≈ 10 days). The mycelia were harvested using sterile distilled water and a cell scraper. This solution was filtered through a cell strainer (70 µm) to obtain a spore-rich suspension, which was diluted to 1 × 10^5^ spores/mL. To 10 µL of spore solution, 10 µL of loaded EVs were added. The solution was carefully mixed and placed on microscope slides, which were kept in a Petri dish with wet filter paper and wrapped in aluminum foil to maintain a humid, dark environment. Samples were left overnight and imaged the following morning. For observation, a Nikon Ni-E fluorescence microscope (Nikon Instruments Inc., New York, NY, USA) was used. NIS-Elements (Nikon Instruments Inc., New York, NY, USA) was used to acquire brightfield and darkfield-illuminated micrographs.

## 3. Results

### 3.1. Callus Induction and Mass Propagation

**Figure 1:**
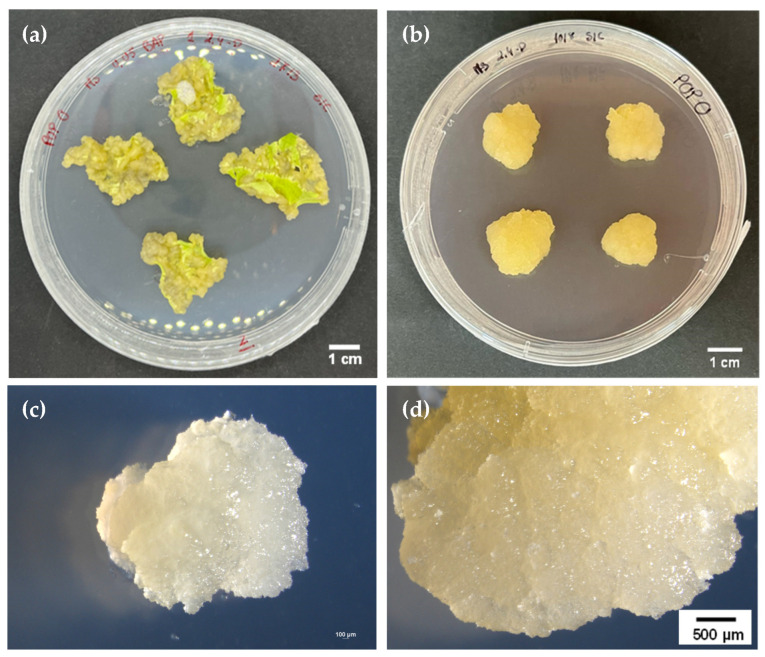
Induced callus from poplar. (**a**) Leaf tissues dedifferentiation in the induction medium after 4 weeks of culture. (**b**) Isolated and proliferating cell line after a 4-week subculture, before EV isolation. (**c**,**d**) Cell line close-up plans, showing the homogenous and friable aspect of the callus with a yellowish colour.

As described, callus was successfully induced in poplar leaf explants [37]. Punctured leaf explants were subcultured 3 times in the same media with intervals of 4 to 6 weeks. Over 17 weeks, these subcultures gradually transitioned through a dedifferentiation phase, during which the tissue progressively lost its original structure and began forming unorganized cell masses (Figure 1a). By the end of this period, a proliferating callus was successfully established (Figure 1b). The resulting callus line was highly homogeneous, friable, and exhibited a distinct yellow coloration, characteristic of active, undifferentiated cells (Figure 1c,d). This line demonstrated vigorous proliferation, requiring transfer to fresh medium every 4 weeks to maintain optimal growth. On average, each culture cycle yielded approximately 500–600 mg of fresh callus tissue, starting from 100 mg, enabling the production of sufficient biomass for extracellular vesicle (EV) isolation.

### 3.2. EV Isolation and Characterization

**Figure 2:**
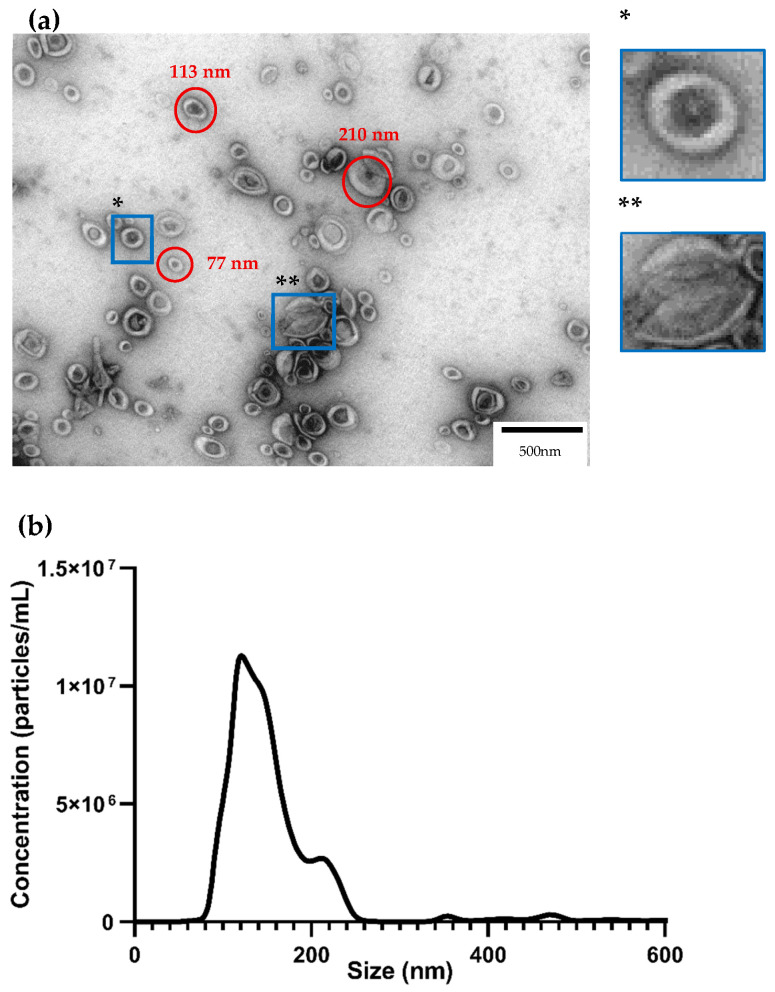
Characterization of the EV population from poplar callus (**a**)—Isolated EVs seen through transmission electron microscopy. * EVs are usually round-shaped with a bilipid layer. ** EVs can collapse and acquire different shapes, such as the common cup shape. (**b**)—Nanoparticle track analysis. The NTA graph shows a big peak at 121 nm and a smaller one at 211 nm. Most of the EVs analyzed were smaller than 200 nm, and the total concentration of EVs in our sample was 2.5 × 10^10^ particles/mL. CS—cup shape; BL—bilipid layer.

Differential centrifugation is a standard method for EV isolation, and it was used in this work to isolate EVs from the callus. This method allows a wide array of vesicles to be isolated, ranging in width from ≈80 to ≈250 nm. Transmission electron microscopy is a powerful tool to confirm the presence of EVs (Figure 2a). Under TEM, EVs often exhibit a distinct bilipid layer and are round; however, the method also causes some EVs to collapse and adopt new shapes. We can observe that some become cup-shaped, and others seem broken. We observed that the EV population in the callus is heterogeneous, with EVs ranging in size from 70 to 220 nm. Aggregation of EVs was observed in most of our images, suggesting that they tend to aggregate into larger blobs. Some smaller structures are sometimes present, though it is difficult to pinpoint exactly what they are due to their irregular shapes. Overall, differential centrifugation worked well to isolate our EVs, with minimal contamination from other sources.

To further characterize our EV population, Nanoparticle track analysis was used. Both TEM and NTA are gold standards in EV characterization, thus, both were employed in this study. NTA allows us to quantify our EV sample and observe the size distribution of the EV population. Two distinct peaks can be observed on the graph (Figure 2b) at 121 nm and 211 nm. The peak at 121 nm is more prominent than that at 211 nm, indicating that these smaller vesicles are the more common type of EVs in our samples. The final concentration of total EVs in our samples was 2.5 × 10^10^ ± 9.6 × 10^8^ particles/mL. The mean size was 142 ± 2.7 nm, the mode was 121 ± 5.0 nm, and the standard deviation was 48 ± 4.2 nm. D10, D50, and D90 were also calculated. The particle size below which 10% of the total particles are contained was 101 ± 3.4 nm, below 50% 132 ± 2.4 nm, and below 90% 191 ± 3.2 nm. This means that the graph area up to 200 nm represents almost all of the EV population in our sample. Some peaks also appear at sizes larger than 211 nm, which may be EV aggregates or machine-induced errors such as out-of-focus particles.

### 3.3. Loading and Uptake Assays

**Figure 3:**
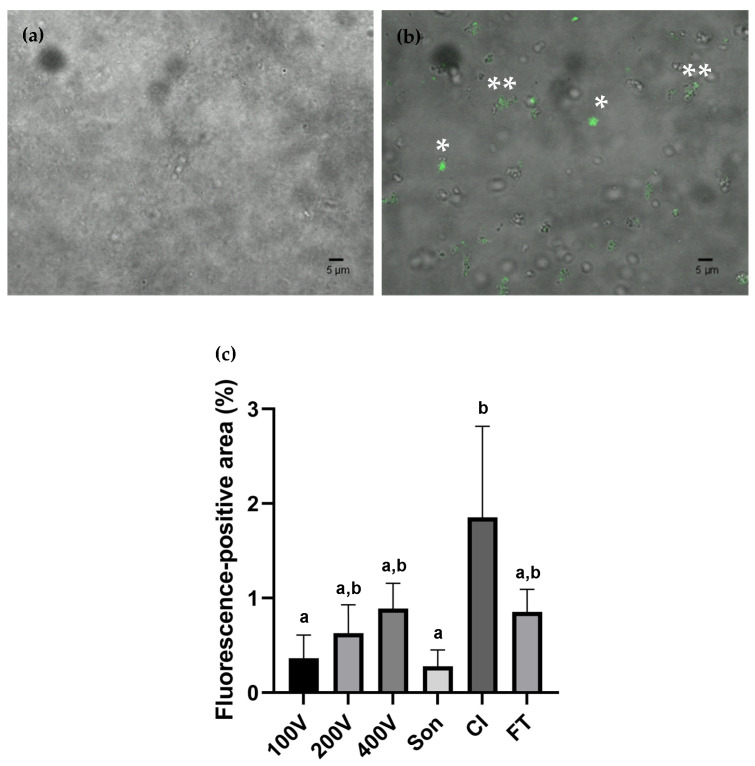
Loading of dsPDS-F into EVs by electroporation (400 V) and uptake by *Botrytis cinerea* spores. (**a**) Confocal overlapped bright field and fluorescent image of unloaded EVs. (**b**) Confocal overlapped bright field and fluorescent images of dsPDS-F-loaded EVs, showing clumps * and dispersed ** loaded EVs. (**c**) Quantification of fluorescently labelled plant EVs after different loading methods. Fluorescence-positive area (%) was quantified from thresholded fluorescence micrographs using ImageJ/Fiji. Loading methods tested were electroporation at 100 V, 200 V and 400 V, sonication (Son), Co-incubation (CI), and freeze–thaw (FT). Data are mean ± SD (*n* = 3). Different superscript letters indicate significant differences (Tukey’s post hoc test, *p* ≤ 0.05).

Different loading assays were performed to assess the loading of fluorescently tagged RNA (dsPDS-F) into isolated EVs: electroporation, sonication, co-incubation, and freeze–thaw. To confirm the effectiveness of these methods, EV samples (Figure 3a) and RNA-loaded EVs (Figure 3b) were treated with MNase to remove any dsPDS-F attached to the outer membrane of the EVs or located outside of the EVs. MNase degraded nucleotides outside the EVs, while those inside the EVs were protected. This confirms that the EVs protected the RNA from MNase. Fluorescence was detected throughout all methods (Figure 3). We observed both dispersed dots and fluorescence agglomerates, likely due to EV aggregation, as shown in Figure 1b.

To assess assay-to-assay loading efficiency, the fluorescence-positive area was measured in ImageJ (Figure 3c). We observed that the loading efficiency was similar across treatments, although significant differences were observed between co-incubation and both electroporation at 100 V and sonication. Electroporation at 400 V compared to 100 V and 200 V shows a slight increase in efficiency.

**Figure 4:**
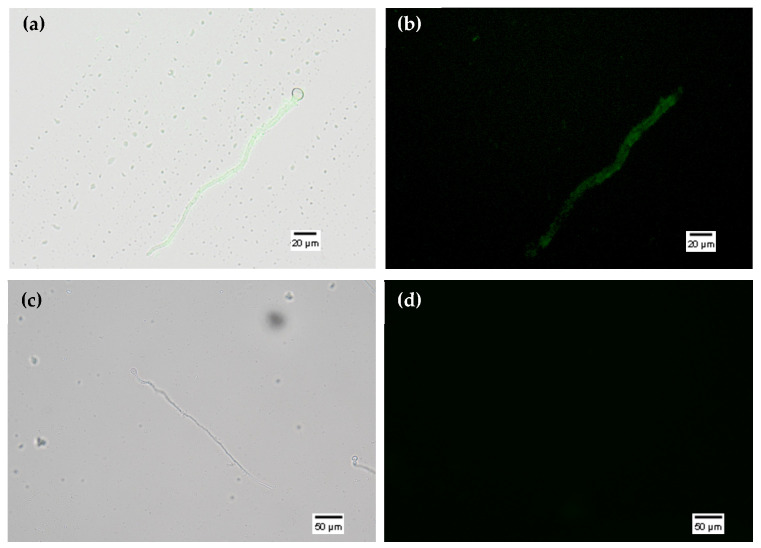
dsPDS-F uptake by *Botrytis cinerea* spores. (**a**) Overlapped bright field and fluorescent images of EP400 EVs incubated with *B. cinerea* spores. (**b**) Fluorescent images of EP400 EVs incubated with *B. cinerea* spores. (**c**) Overlapped bright field and fluorescent images of sonicated EVs incubated with *B. cinerea* spores. (**d**) Fluorescent images of sonicated EVs incubated with *B. cinerea* spores. Example of no uptake observed.

Following confirmation that all assays resulted in dsPDS-F-loaded EVs, these were used to perform uptake assays in *Botrytis cinerea* spores. This is a reliable system for observing RNA uptake of single- and double-stranded RNAs of different sizes. dsPDS-F-loaded EVs were incubated with germinating spores, and fluorescence was observed with an epifluorescence microscope (Figure 4). Germinating *B. cinerea* spores incubated with EVs electroporated at 400 V (EP400) with dsPDS-F showed fluorescence after 16 h (Figure 4a,b), indicating that the RNA-loaded EVs delivered their cargo into the hyphae. Despite being able to load dsPDS-F into the EVs using different methods, we observed dsPDS-F uptake by *B. cinerea* hyphae only when loaded by electroporation (400 V). When dsPDS-F was loaded into EVs via sonication, co-incubation, freeze–thaw, and electroporation at 200 and 100 V, we did not observe uptake by the hyphae (Figure 4c,d). It is also worth noting that fluorescence was detected only in the hyphae, never in the spores.

## 4. Discussion

Poplar cell lines are ideal to study not only somatic embryogenesis [38], but also to explore how they can be a tool in current and future systems [39,40]. The ability to produce these in bioreactors enables us to mass-produce desired biomolecules, such as secondary metabolites and enzymes [41], in a safe environment. The same could be applied to the mass production of EVs. This would allow us to obtain safe, clean EVs for biotechnological and medical applications. To further demonstrate that these could be a desirable system for EV production, it is possible to genetically engineer these cell lines to produce EVs with the desired characteristics [42]. In sum, by combining mass production and bioengineering of plant cell lines, we can obtain the desired EVs in a clean and safe environment.

Studies of plant cell line-derived EVs are recent, but they have already shown some potential [5,6,7,8,9,43]. Most of these aimed to characterize EVs from different types of cell lines. EVs from a Norway spruce cell line were suggested to be involved in the transport of enzymes responsible for lignin formation [5]. EVs from an *Arabidopsis thaliana* cell line contained proteins important for cell wall biogenesis and pathogen defence mechanisms [8]. EV characterization and their uptake by plant and rat cells were achieved in a tobacco cell line [9]. This study was followed by the characterization and loading of EVs from different sources, namely, callus, cell liquid culture, and leaf apoplast [43]. Kırbaş and colleagues (2024) isolated EVs from cell suspensions of *Stevia rebaudiana* and *Vaccaria hispanica*. These were characterized, and an uptake assay was performed, where these were successfully taken up by T lymphocyte cell lines after 2 h incubation [6]. EVs from ginseng showed potential to enhance skin regeneration and were suggested as candidates for drug delivery in therapeutics [7]. From all these studies, one common conclusion was that EVs from cell lines can potentially be up-scaled and mass-produced for biotechnological applications.

As observed in TEM imaging (Figure 2a), these are nano-sized structures with a pronounced bilipid layer and are very similar to those found in other plant EVs [8,30]. Sometimes artefacts may appear due to TEM sample preparation or the EV isolation method, such as the cup-shaped appearance of some imaged EVs rather than their original round shape [14]. We also noticed that sometimes other structures were present, which could be caused by debris, image noise, dye remnants, or co-isolated small protein complexes or lipoproteins [44,45]. EVs are also known to aggregate in big clumps, as observed in Figure 2b. This could lead to erroneous readings during NTA, hence the need to characterize EVs through TEM. As shown by Kocholatá and colleagues (2022), to avoid these artefacts, trehalose can be added to the EV sample to separate the EVs [9]. As for size, the produced EVs have a mean size of 142 nm, similar to that observed in Norway spruce cell lines, where most EVs were below the 150 nm threshold [5]. Moreover, the mean sizes of *Arabidopsis thaliana* [8], ginseng [7], and tomato [9] were closer to 200 nm, and the populations appear highly heterogeneous. These differences may be due to the methodologies employed in the studies or to the organisms being studied, which may differ in their physiology and EV secretion [7].

In the present work, we observed that our callus produced 2.5 × 10^10^ particles/mL. In tobacco, it was observed that isolated callus EVs accounted for 1.95 × 10^10^ [43]. They also observed that suspension cultures had 5.70 × 10^9^ and apoplastic fluid from leaves, 1.24 × 10^9^ [43], which are well below the numbers obtained from callus cultures. Another example is from EVs isolated from *Arabidopsis thaliana* callus, which yielded 1.8 × 10^10^ particles/g [8]. These results are very similar to ours, confirming that this is a sound system for EV isolation from a safe, clean source.

*Calli* are known for their high proliferation and enhanced metabolic activity [46,47,48,49], which can be good indicators of high EV production. Furthermore, callus induction and growth are subjected to stress, whether from plant growth regulators or abiotic stress [3], which are known to induce EV production [30]. In sum, high proliferation, enhanced metabolic activity, and stress exposure are effective mechanisms for achieving high EV production, making poplar callus a promising system for EV production.

Although EV production from poplar cell lines offers considerable advantages, scaling this process for larger–scale applications remains a critical challenge. In the present work, EVs were isolated from callus cultured on solid media; however, the adoption of liquid culture systems could also facilitate large-scale EV recovery [6,8,9].

In the future, it would be important to test the growth of poplar cell lines in liquid media (e.g., in a bioreactor), followed by EV extraction, as it would be much easier to handle and harvest from a bioreactor with litres of media and growing cell suspensions. Considering space, feasibility, handling, and time [43], we think growing cell suspensions in bioreactors is the most objective way for upscaling.

Loading methods are highly dependent on the size of RNA or DNA molecules [50]. siRNAs and microRNAs (miRNAs) can be easily loaded via sonication, co-incubation, electroporation, and other methods [51]. Larger molecules, of sizes between 100 and 250 bp, seem to be loaded through electroporation and specialized methods, although their efficiency is not significant [52]. Bigger RNAs like mRNAs need more specialized methods and advanced loading techniques [53]. In our case, the RNA is around 200 bp, which could explain why our methods had low RNA loading. In cases of EVs loaded by sonication, co-incubation, freeze–thaw, and electroporation at 100 V and 200 V, there was no RNA taken up by the hyphae. An explanation for why no RNA from these methods entered the fungal cells is hard to find, except for the EVs electroporated at 400 V. We showed that the fluorescent RNA was protected from MNase, but despite that, we did not see any fluorescence during the uptake assays. In these cases, the RNA may have been unable to load into the EVs but remained protected in the EV corona, providing protection for the RNA. Subsequently, during uptake assays, the low amount of RNA within the EVs’ corona could have been dispersed and lost, thus not providing a positive result. Further optimization of these methods is required to yield better results and ensure repeatability, and should therefore be pursued.

Electroporation at 400 V produced one of the best results in both loading and uptake by fungal hyphae. This further corroborates that the hyphae are responsible for the RNA internalization from the previously loaded EVs [28]. The RNA uptake by the fungal cell is very interesting, as it suggests that EVs from poplar cell lines could be used to protect, transport, and deliver RNA molecules of this size (≤220 bp), and probably siRNAs and miRNAs, which are smaller and easier to load. It was demonstrated that smaller RNAs could be loaded into EVs from a tomato cell line, through long incubation and sonication [43], whereas electroporation was the least effective method.

Overall, the results show some promise that poplar EVs can be loaded with RNA, protect their contents, and deliver their cargos into fungi hyphae. Despite the results, further experiments should include loading smaller RNAs into poplar EVs and verifying if these can be taken up by fungal cells. Going even further, the effectiveness of such RNAs should be assessed after loading into EVs, since it may be compromised by the loading method, retention within EVs, or failure to deliver to targets [52,54].

When testing EV-based products, a systematic evaluation of EV toxicity is an essential aspect for their practical application in plant protection. A key aspect of callus-derived EVs is their low cytotoxicity and high stability, which are key features of EV-based solutions [17,43]. Despite their low toxicity to both plants and humans, they can still be toxic to insects and fungi [55]; therefore, a study on the effects of poplar callus-derived EVs on such pests could be worthwhile exploring.

As new techniques are emerging in the RNAi field for crop protection [28], it is also time to consider how we can improve their stability and effectiveness. siRNAs and RNAs, in general, are prone to degradation in the environment and foreign organisms [24], and if we wish to employ them in crop protection, their stability must be improved. EVs are reliable nanocarriers for the efficient protection and delivery of RNA, are low-toxicity, and should be considered strong contenders for improving RNAi techniques.

## 5. Conclusions

In this study, we show that extracellular vesicles (EVs) from poplar cell lines can be efficiently isolated at relatively high yields, reaching ~2.5 × 10^10^ particles/mL. Their nanoscale size, stability, and natural capacity to transport biomolecules support their potential as delivery vehicles for RNAi and related applications. We successfully loaded poplar EVs with fluorescently labelled dsRNA via electroporation, and our results indicate that other loading strategies, particularly those using smaller RNAs, may also be viable with further optimization. Although overall loading capacity was similar across treatments, 100 V electroporation and sonication performed the worst.

EVs loaded through 400 V electroporation delivered RNA that was taken up by fungal cells, emphasizing the importance of testing whether shorter RNAs or other cargo types show comparable uptake. Future work should therefore focus on evaluating the functionality of EV-delivered RNAs and clarifying how EV–fungus interactions influence RNA stability, availability, and delivery efficiency.

Because RNA quantification was not carried out during loading or uptake, establishing quantitative assays will be essential, along with improving data repeatability. Determining RNA loading capacity will enable a more accurate comparison of loading methods, while quantifying uptake (e.g., via fluorescent hyphae counts) will strengthen the development of EV-based delivery systems. Additional priorities include assessing the long-term stability of RNA-loaded EVs, the persistence of the RNA cargo, and the effects of EV aggregation on loading efficiency and uptake.

Overall, this work advances the characterization and biotechnological potential of plant-derived EVs and highlights their promise as versatile carriers for future RNA-based interventions.

## Figures and Tables

**Table 1:** Primers used for IVT (underlined bases: T7 RNA Polymerase promoter).

Primer Name	Sequence (5′–3′)
TaPDS Frw	TTTGCTCCAGCAGAGGAATGG
TaPDS Frw T7pol	TAATACGACTCACTATAGGTTTGCTCCAGCAGAGGAATGG
TaPDS Rev	AAACCCTTCGATCGGTGATCG
TaPDS Rev T7pol	TAATACGACTCACTATAGGAAACCCTTCGATCGGTGATCG

## Data Availability

The original contributions presented in this study are included in the article. Further inquiries can be directed to the corresponding author.

## Abbreviations

The following abbreviations are used in this manuscript:

2,4-D	2,4-dichlorophenoxyacetic acid
AWF	Apoplastic Washing Fluid
BAP	6-Benzylaminopurine
miRNA	micro RNAs
MNase	Micrococcal Nuclease
NTA	Nanoparticle Track analysis
RNAi	RNA interference
siRNA	small interfering RNAs
TEM	Transmission Electron Microscopy
VIB	Vesicle Isolation Buffer
